# Thermal sensitivity links to cellular cardiac decline in three spiny lobsters

**DOI:** 10.1038/s41598-019-56794-0

**Published:** 2020-01-14

**Authors:** Michael Oellermann, Anthony J. R. Hickey, Quinn P. Fitzgibbon, Greg Smith

**Affiliations:** 1 Fisheries and Aquaculture Centre, Institute for Marine and Antarctic Studies (IMAS), University of Tasmania, Hobart, Tasmania 7001 Australia; 2 School of Biological Sciences, University of Auckland, Auckland, New Zealand

**Keywords:** Cell biology, Ecology, Physiology, Zoology

## Abstract

Understanding mechanisms of thermal sensitivity is key to predict responses of marine organisms to changing temperatures. Sustaining heart function is critical for complex organisms to oxygenate tissues, particularly under temperature stress. Yet, specific mechanisms that define thermal sensitivity of cardiac function remain unclear. Here we investigated whole animal metabolism, cardiac performance and mitochondrial function in response to elevated temperatures for temperate, subtropical and tropical spiny lobster species. While oxygen demands increased with rising temperatures, heart function became limited or declined in all three species of lobsters. The decline in cardiac performance coincided with decreases in mitochondrial efficiency through increasing mitochondrial proton leakage, which predicts impaired compensation of ATP production. Species differences were marked by shifts in mitochondrial function, with the least thermal scope apparent for tropical lobsters. We conclude that acute temperature stress of spiny lobsters, irrespective of their climatic origin, is marked by declining cellular energetic function of the heart, contributing to an increasing loss of whole animal performance. Better understanding of physiological thermal stress cascades will help to improve forecasts of how changing environmental temperatures affect the fitness of these ecologically and commercially important species.

## Introduction

Temperature is one of the major driving forces shaping survival and physiological performance of marine organsims^1^. Particularly in shallow coastal and intertidal waters, organisms require to cope with frequent changes of temperatures across space and time. Outcomes of acute or chronic thermal stress can be significant, not only to individuals but whole populations, leading to large scale re-distribution, decline or even collapse of local populations^2–4^. Lobsters are ecological key players, both as predators and prey^5^ and crucial to sustain ecosystem resilience by e.g. buffering destructive species shift^6^. They are one of the most highly valued marine food sources^7,8^ and promising candidates for an emerging global aquaculture industry^9,10^. However, lobsters are particularly exposed to temperature changes, due to their inability to regulate body temperature (ectothermic) and the distribution of numerous species in shallow coastal waters, where temperatures vary naturally during tidal cycles, strong winds or across thermoclines by more than 10 °C^11,12^. For some species, thermal limits have been exceeded in some areas^13–16^, which causes concern given that ocean are predicted to warm on average by 2.7 °C by the end of this century (from 1990, RCP8.5^17^). An increasing frequency, intensity and duration of heat waves and localised above average warming trends^18,19^, will likely exacerbate long term trends, and may pose acute risks to local populations with small thermal safety margins^16,20^. Understanding when and why lobsters reach thermal limits will improve mechanistic models to predict the wellbeing of future populations^21–23^, as well as inform protective management actions to e.g. identify and preserve thermal refugia^24–26^.

The mechanisms underlying temperature limitation of marine ectotherms remain debated, as these may vary, depending on a species physiology, life stage (e.g. larvae vs adult), physiological state (e.g. age, disease, growth, reproduction), and on intensity and exposure (acute or chronic) of temperature stress^27^. Oxygen limitation has been proposed as primary factor in limiting thermal tolerance in aquatic ectotherms^28,29^. Accordingly, marine ectotherms increasingly struggle to sustain adequate tissue oxygen levels when temperatures increase, due to an inability of the ventilatory and cardiovascular system to match the steady increase of cellular oxygen demands^30^. This concept remains controversial with support for and against e.g.^31–34^. One reason may be the lack of detailed understanding if and how different components of the oxygen delivery system may trigger a cascade of thermal failure^27,35^.

The mechanisms of thermal limitation are still poorly understood for crustaceans. An impaired response to increase of ventilation with rising temperature, and a coincident declining arterial oxygenation have been suggested to be significant factors that combine to limit oxygen supply at high temperatures^33^. In contrast, the role of cardiac performance in thermal limitation was proposed to be minor for most crustaceans^33^. However, this interpretation is limited to heart rate measures as proxies of performance^36–39^. Indeed, other cardiac performance measures cease to rise and compensate for rising temperatures, or even decrease. These include cardiac stroke volume^12,40–42^, cardiac output^12,43^, or cardiac contraction force^12^. While this may be linked to decreased arterial oxygen supplies to cardiac tissues, crustacean hearts - unlike most teleost hearts^44^ - are less likely to become oxygen depleted as their hearts receive oxygenated blood directly from the gills. Further, control by cardio-regulatory nerves and neurohormones arising from other parts of the central nervous system are also unlikely to drive cardiac temperature dependence, given that similar thermal sensitivities of hearts are apparent *in vivo* and *ex vivo*^12^. This indicates that some intrinsic factors mediate the temperature dependence of cardiac performance. The cardiac ganglion is the prime candidate for control of the crustacean neurogenic heart^45^, however, alternative intrinsic mechanisms that define thermal sensitivity have not been well considered. Mitochondria present one such target within cardiac tissues and are well known to respond to rising temperatures through a decline in oxidative phosphorylation efficiency through impaired inner mitochondrial membrane integrity^46–49^.

To date the relation between the thermal sensitivity of mitochondrial function and cardiac performance has been poorly assessed in crustaceans. So far it has only been tested by comparison of two species of portunid crab, but not for other crustaceans of diverse climatic origins^49^. It is also unclear whether patterns of thermal sensitivity remain consistent across closely related species of lobsters that have differing thermal histories (i.e. thermal adaptation) or un-linear change occurs, as the case for distinct populations of European green crabs^50^.

**Figure 1:**
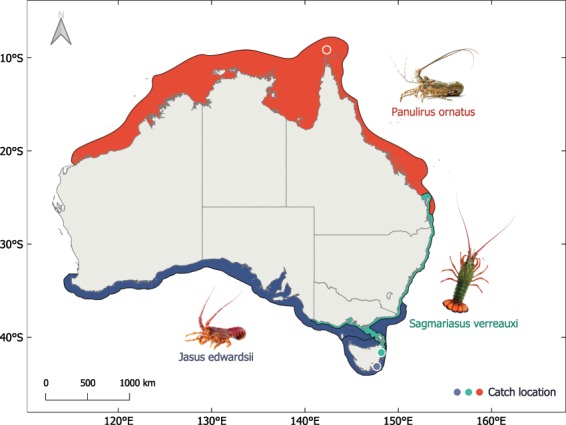
Geographic distributions of *J. edwardsii*, *S. verreauxi* and *P. ornatus*. Data sources: Atlas of Living Australia, Redmap.org. Catch location marked by coloured circles. Image courtesy of Craig Mostyn Group and Seafood New Zealand.

We compared three spiny lobsters species from different thermal habitats, (1) the cold-temperate to temperate *Jasus edwardsii*, (2) the subtropical *Sagmariasus verreauxi* and (3) the tropical *Panulirus ornatus* (Fig. 1). We then tested (1) how whole animal metabolism and cardiac performance respond to acute increases in temperature, (2) if cardiac thermal sensitivity mirrors cardiac mitochondrial function and (3) if there are universal and species-specific patterns that dictate thermal sensitivity or cardiac performance.

Combined whole animal respirometry and heart rate logging was undertaken, followed by measurements of cardiac mitochondrial energy production. This revealed a species-independent divergence between increasing oxygen demand and limited or declining cardiac performance with increasing temperatures. This paralleled decreasing cardiac mitochondrial stability of respiratory complex I and mitochondrial membrane stability, where the latter resulted in decreased energetic efficiency in terms of work done to maintain a membrane potential, and ATP production. The tropical spiny lobster showed the greatest response in mitochondrial function with increasing temperature, and the least margin between optimal and critical cellular temperatures.

## Results

### Thermal divergence between oxygen demand and cardiac performance

**Figure 2:**
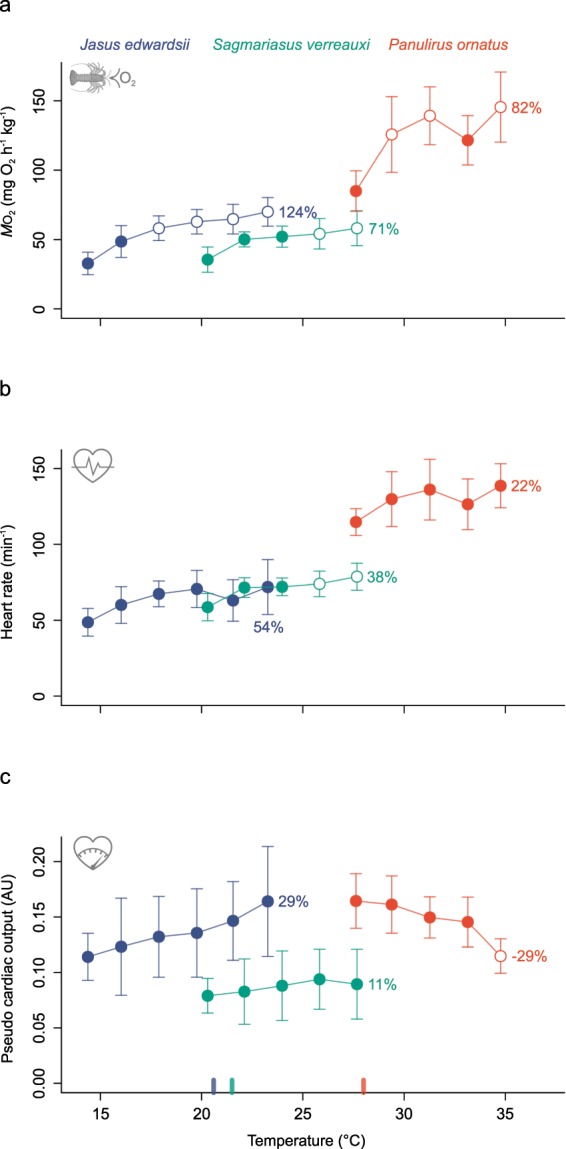
**(a)** Change of total oxygen consumption, **(b)** heart rate and **(c)** pseudo cardiac output (expressed in arbitrary units AU) with increasing temperatures for three spiny lobster species - cold-temperate *J. edwardsii* (blue), subtropical *S. verreauxi* (green) and tropical *P. ornatus* (red). Data presented as means ±95% CI, *n* = 6–8. Open circles indicate significant difference relative to the lowest temperature for each species. Percentage values indicate relative change between starting and final temperature. Coloured tick marks at bottom x-axes indicate optimal temperatures for growth corresponding to each species (20.6 °C for *J. edwardsii*^100^, 21.5 °C for *S. verreauxi*^34^ and 28 °C for *P. ornatus*^82^).

**Figure 3:**
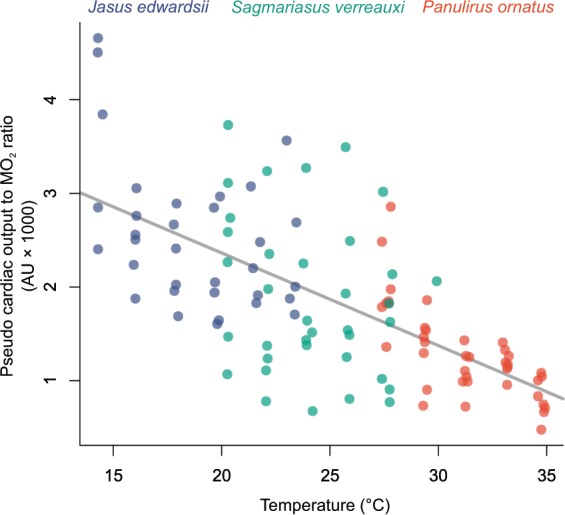
Pseudo cardiac output decreases linearly relative to spiny lobster oxygen consumption with increasing temperatures (y = −0.10 × +4.34). A linear regression model was applied to the pooled data set, as a more complex model with species as co-factor did not significantly improve the linear regression model (ANOVA (*F*_(4, 107)_ = 0.60, *P* = 0.67).

Simultaneous measurements of whole animal oxygen consumption and heart rate revealed a growing gap between increasing demand for oxygen and plateauing or declining cardiac performance with acutely rising temperatures (Fig. 2). While oxygen consumption increased in all three species with increasing temperature (Fig. 2a, 14–24 °C for *J. edwardsii*, 20–28 °C for *S. verreauxi*, 27–35 °C for *P. ornatus*), heart rate did not increase significantly in *J. edwardsii* and *P. ornatus*, except for *S. verreauxi* (Fig. 2b). Pseudo cardiac output did not increase significantly with increasing temperature in any of the three species and declined significantly in *P. ornatus* beyond 33 °C (Fig. 2c). More clearly, heart rate and pseudo cardiac output changed at much lower rates than oxygen consumption relative to their initial starting temperature (Fig. 2a–c, refer to percentage values), with a maximal gap between relative change of oxygen consumption (82% ± CI 54%) and pseudo cardiac output (−29% ± CI 10%) in *P. ornatus* (Fig. 2a,c). Ratios of pseudo cardiac output to oxygen consumption rate highlighted a general decrease of pseudo cardiac output relative to the total oxygen utilised by the animal with increasing temperature (Linear regression *F*_(1,107)_ = 87.15, *P* < 0.01, R^2^_adj._ = 0.44, Fig. 3).

### Decline of cardiac performance

**Figure 4:**
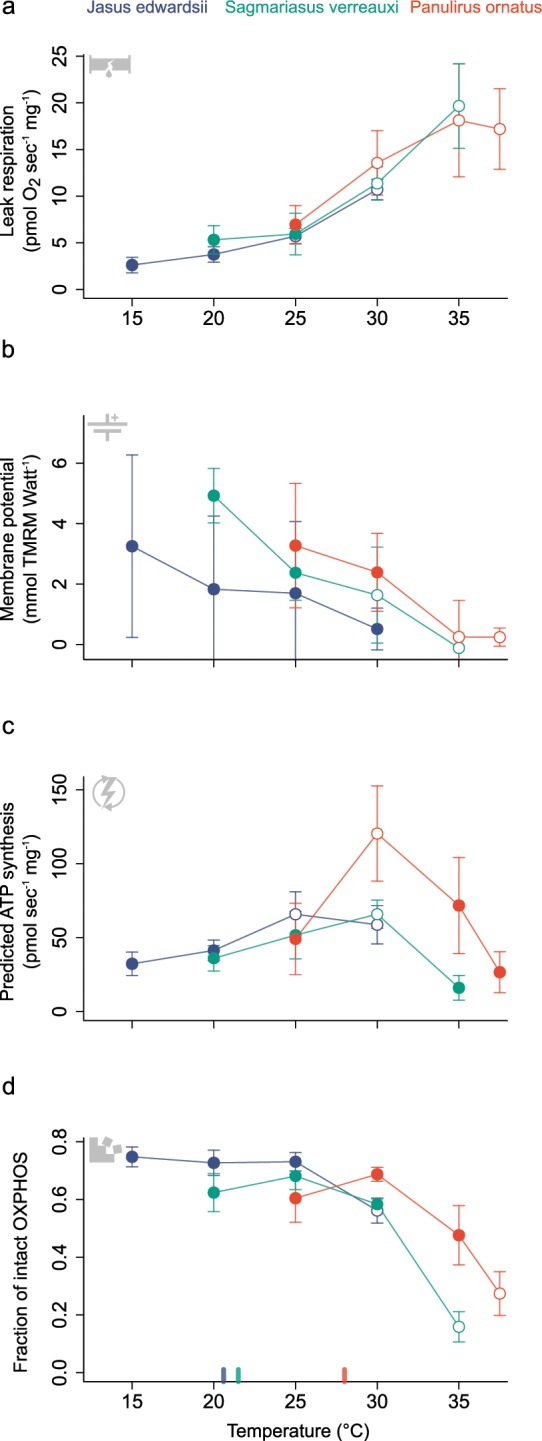
Cardiac tissue change of **(a)** leak respiration (following addition of complex III inhibitor oligomycin), **(b)** mitochondrial membrane potential, **(c)** predicted ATP synthesis rates and **(d)** fraction of intact mitochondrial ATP production (i.e. oxidative phosphorylation (state 3 respiration - leak respiration)/state 3 respiration) with increasing temperatures in comparison between cold-temperate *J. edwardsii* (blue), subtropical *S. verreauxi* (green) and tropical *P. ornatus* (red). Data presented as means ±95% CI, *n* = 6–8. Open circles indicate significant difference relative to the lowest temperature for each species. Coloured tick marks at bottom x-axes indicate optimal temperatures for growth corresponding to each species (20.6 °C for *J. edwardsii*^100^, 21.5 °C for *S. verreauxi*^34^ and 28 °C for *P. ornatus*^82^).

**Figure 5:**
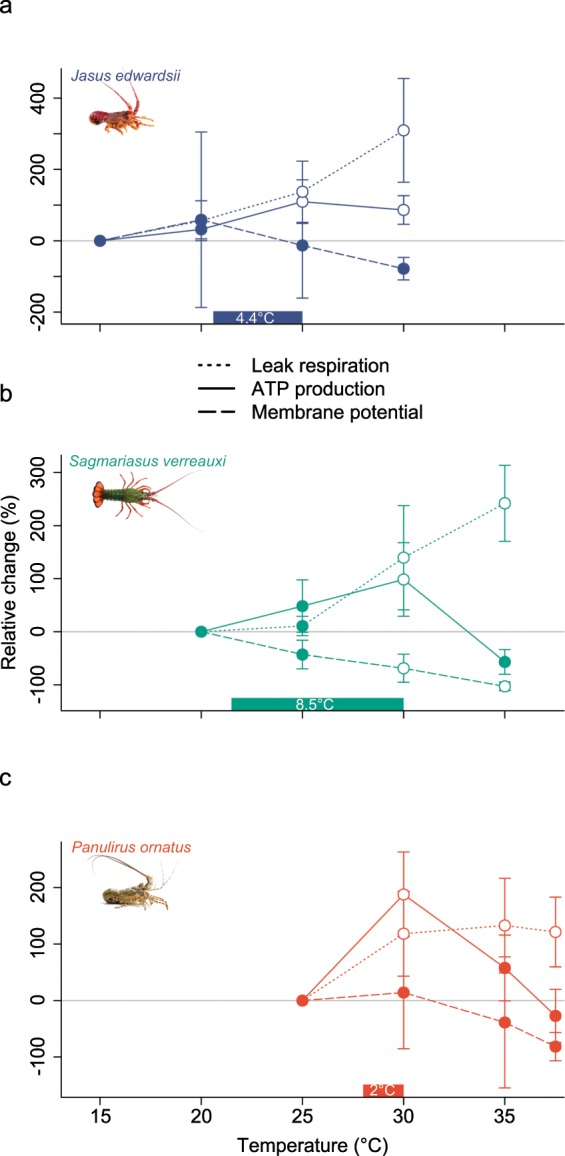
Relative change (%) of spiny lobster cardiac leak respiration (dotted line), ATP production (solid line) and membrane potential (dashed line) in comparison between **(a)** *J. edwardsii* (blue), **(b)** *S. verreauxi* (green) and **(c)** *P. ornatus* (red). Data presented as means ±95% CI, *n* = 6–8. Open circles indicate significant difference relative to the lowest temperature for each species. Coloured bars indicate margins between optimal temperature for growth and temperatures, at which leak respiration and ATP production start misaligning. Image courtesy of Craig Mostyn Group and Seafood New Zealand.

Subsequent mitochondrial assays on cardiac tissues of the experimental lobsters revealed declining efficiency of aerobic energy release by cardiac mitochondria in all three lobsters with increasing temperatures (Fig. 4). Independent measurements of mitochondrial leak respiration (i.e. state III respiration using oligomycin) and membrane potential (i.e. fluorescence of membrane selective TMRM) showed that proton leakage across the inner mitochondrial membrane into the matrix space likely increases with increasing temperature (Figs. 4a,b, 5a,b). Estimates of mitochondrial production of the main energy carrier ATP followed an optimum temperature pattern, with predicted ATP production peaking at 25 °C for *J. edwardsii*, 30 °C for *S. verreauxi*, and 30 °C for *P. ornatus* (Figs. 4c, 5a–c). Below peak temperatures for ATP production, mitochondrial proton leak and ATP production increased at similar levels, but then diverged beyond optimal temperatures, with proton leak increasing further and ATP production plateauing or rapidly declining (Fig. 5a–c). As a result, mitochondrial capacity to produce ATP likely decreases precipitously above optimal temperature (i.e. intact fraction of oxidative phosphorylation, Fig. 4d).

### Species trends and climate origin

Thermal sensitivity of spiny lobster from different geographic and climate origins revealed similar patterns of cardiac performance, yet also species-specific characteristics. All three species displayed declining pseudo cardiac output relative to whole-animal oxygen consumption (Fig. 3). This trend was similar among species and suggests similar responses regardless of climatic/thermal habitat origin (i.e. linear regression models for each single species did not perform better than linear regression for the pooled data set, ANOVA, *F*_(4,103)_ = 0.59, *P* = 0.67, Fig. 3). Similarly, mitochondrial leak respiration increased significantly with temperature (Linear regression, *F*_(1,81)_ = 155.89, *P* < 0.01, R^2^_adj._ = 0.65) and this pattern was indistinguishable among species (i.e. linear model comparison pooled vs. species data, ANOVA, *F*_(4,77)_ = 1.49, *P* = 0.21, Fig. 4). Membrane potential also decreased similarly in all three species (Linear regression with temperature as co-factor, *F*_(5,74)_ = 10.52, *P* < 0.01, R^2^_adj._ = 0.38), however, there was a significant temperature offset between *J. edwardsii* and *S. verreauxi* (*P* = 0.01).

**Figure 6:**
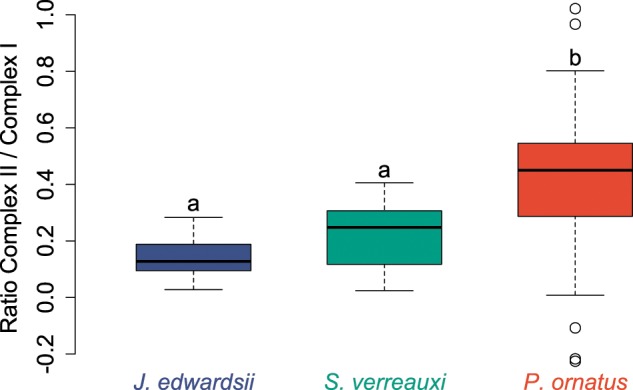
The ratio of mitochondrial complex II respiration (succinate + ADP) to complex I respiration (complex I substrates + ADP) increases from temperate *J. edwardsii*, subtropical *S. verreauxi*, to tropical *P. ornatus*. Letters indicate significant differences between species (Chi square = 23.3, *P* < 0.01, *df* = 2). Data were pooled for each species, due to lack of significant differences among experimental temperatures.

Differences among species were most pronounced for the tropical rock lobster, *P. ornatus*. While predicted ATP production was similar among all lobsters at 25 °C, the rates of *P. ornatus* tripled at 30 °C, exceeding the plateauing rates observed for *J. edwardsii* and *S. verreauxi* by 1.8–2.0 times. Accordingly, unlike for *J. edwardsii* and *S. verreauxi*, oxidative phosphorylation in *P. ornatus* remained intact up to 30 °C, and then decreased due to a steep decline of predicted ATP production (Fig. 4c,d). Notably, *P. ornatus* showed the smallest margins between optimal temperatures for growth and temperatures at which relative changes of cardiac leak respiration and predicted ATP production started to diverge (2 °C in *P. ornatus* versus 4.4 °C for *J. edwardsii* and 8.5 °C for *S. verreauxi*, Fig. 5). Lastly, the contribution of mitochondrial complex II to ATP production was 2–3 times higher in *P. ornatus* compared to *J. edwardsii* and *S. verreauxi* respectively (Fig. 6).

## Discussions

Our data demonstrated a key role of declining cardiac mitochondrial function in defining thermal sensitivity of spiny lobsters. Successive increases in mitochondrial proton leak alongside limited compensation in predicted ATP production rates indicates an increasing mismatch of cardiac performance and whole animal oxygen consumption, irrespective of whether lobsters originated from cool-temperate, subtropical or warm-tropical waters.

Cardiac performance in Australian spiny lobsters increasingly diverges with increasing whole-animal oxygen consumption when temperatures rise acutely, indicating a role of cardiac limitation in thermal tolerance. This was supported by declining pseudo cardiac output relative to individual oxygen demand, irrespective of species or their climate origin (Fig. 3). By contrast, a continuous increase of heart rate but declining ventilation capacity prior to critical temperatures has been reported for several crustacean species, and it was suggested that cardiac performance is of minor importance in limiting thermal tolerance as opposed to branchial oxygen uptake^33^. However, many studies solely used heart rate as performance proxy, without including cardiac stroke volume to estimate overall cardiac output^36–39^. In fact, in many crustaceans, cardiac stroke volume plateaus or decreases with increasing temperature, inversely to heart rate, thus reducing or offsetting a thermal increase of cardiac output^12,40–43,51^. Wooden *et al*.^12^ explained this discrepancy by an independent control of heart rate and the heart beat strength in response to temperature. Heart rate is directly paced by the cardiac ganglion motorneurons^12,45^ and may also affect stroke volume indirectly, by defining the available time period for the heart to relax and contract to reach its full pump volume^52^. However, cardiac contraction strength and dependent stroke volume are uncoupled from heart rate in American lobsters, indicating that the overall cardiac output, results from the thermal sensitivities of two independent mechanisms^12^. Consequently, despite increasing heart rate, overall cardiac output and thus circulatory oxygen supply may indeed become limiting in crustaceans prior to critical temperatures.

The observed lower thermal increase of heart rates relative to oxygen consumption rates in all three species (Fig. 2b), further indicates divergence between cardiac performance and oxygen demand at higher temperatures, but also indicates some oxygen buffering mechanism, or increased dependence on anaerobic capacities. Intuitively, the relative change of heart rates is expected to align with oxygen consumption to match blood oxygen supply with increasing oxygen demands. However, thermal sensitivities of heart rates are highly variable among species or life stage, either being less^40^, similar^36,37^ or greater in response to relative changes in oxygen consumption rate^51^. A lower thermal increase of heart rate - as observed in this study - may lead to an oxygen deficit if there is no compensation. This could be achieved by increasing stroke volume, which however, plateaued or declined in all three spiny lobsters, indicating the presence of other compensatory mechanisms, or limitations on the heart. First, unlike primitive crustaceans, decapod crustaceans can directly control the flow of haemolymph to different parts of their body via seven arteries exiting the heart, each controlled by cardio-arterial valves^53,54^. Dungeness crabs redistribute their haemolymph flow when temperatures increase^41^ and this is more pronounced during hypoxia, and may act to preserve oxygen for critical organs such as ventilatory muscles of scaphognathites^55^. Spiny lobster too may selectively regulate cardiac haemolymph efflux to organs when temperatures rise, to lower systemic oxygen demand and consequently cardiac pumping, resulting in a lower thermal increase of heart rates. This may also explain selective organ damage, such as of crustacean´s locomotor muscle, which suffer from reduced mitochondrial membrane potential and aerobic fibre area^56^, following severe temperature or hypoxic stress. Second, the blood pigment haemocyanin has been shown to support blood oxygen supply by increased unloading at higher temperatures, thus decreasing demands for cardiac pumping^40,43,57^. Lastly, physiological structures differ between species and life stage and may alter cardiac oxygen supply. For instance, millimetre-sized crustaceans or larvae are able to supply peripheral tissues by diffusive oxygen uptake directly via the exoskeleton, thus decreasing the dependence on convective oxygen transport, particularly when ambient-tissue oxygen gradients are steep^58–60^. Overall, the combination of dampening of elevation of the heart rate with rising temperatures, and a stable or decreasing pseudo cardiac output indicate increasing cardiac limitation at higher temperatures in spiny lobsters, where buffering mechanism (e.g. selective haemolymph distribution, haemocyanin oxygen reserve, cutaneous respiration) may sustain circulatory oxygen supply for short durations.

Most importantly, our results demonstrated that thermal sensitivity of cardiac performance in all three species of spiny lobsters links to declining cellular cardiac function, due to an increase of leak respiration and a decrease in mitochondrial membrane potential when temperatures rise (Fig. 4). Therefore, aerobic ATP production by cardiac mitochondria becomes increasingly inefficient, as energy (i.e. the proton gradient) dissipates across the inner mitochondrial membrane back into the mitochondrial matrix, without being utilised for ATP production. We note that the estimated ATP production initially increases in parallel with leak respiration, and this should largely compensate for this loss of energy, although will incur increased energetic costs in terms of substrate oxidation and oxygen uptake. However, at 25 °C for *J. edwardsii* and 30 °C for *S. verreauxi* and *P. ornatus*, ATP production plateaus or declines (Figs. 4c, 5), while leak respiration continued to increase (Figs. 4a, 5). This likely marks a critical temperature beyond which cardiac mitochondrial energy production must deteriorate. Temperature dependent increases in proton leak and resulting loss of phosphorylation capacity is a frequently observed phenomena^46,47,49,61,62^ and is related to increases of membrane permeability^63^ and proton flux via mitochondrial membrane proteins such as uncoupling proteins (in mammals)^48^, dicarboxylate carriers^64^, the adenine nucleotide translocase^65,66^, or aspartate/glutamate antiporter^67^. Therefore, despite an increasing uptake of metabolic energy (e.g. carbohydrates) and oxygen, mitochondria may become increasingly unable to supply sufficient ATP to power cardiac muscle contraction in spiny lobsters. Although not measured in this study, cardiac contraction strength declines along with stroke volume in heat stressed American lobster heart^12^, suggesting that in part a declining energy supply by mitochondria to cardiac muscle fibres contributes to declining stroke volume as temperature increases. In addition, this finding could present an intrinsic cardiac mechanism that in part could contribute to thermal sensitivity of cardiac output, which is independent from the thermal sensitivity of the ganglion controlled heart rate.

The comparison of three species of spiny lobster from distinct climate origins revealed general trends of cardiac and cellular performance. All three species displayed decreasing energetic efficiency of cardiac mitochondria (i.e. increasing proton leak, Fig. 4a,b), indicating a functional link with the general decline of pseudo cardiac output relative to total oxygen demand (Fig. 3). Therefore, for spiny lobsters, loss of energetic efficiency poses an underlying thermal challenge, compromising cardiac performance under acute as well as chronic sub-lethal temperature stress. This may be particularly critical during periods of high metabolic demand such as locomotion, moulting, reproduction or digestion. Loss of energetic efficiency, may not be exclusive to hearts but likely applies to other aerobic tissues as well, causing a disproportional increase of overall energy demand and diversion of energy away from essential processes such as growth, reproduction or locomotion^47,68^.

Our data also highlighted marked differences among species. Unlike temperate *J. edwardsii* and subtropical *S. verreauxi*, tropical *P. ornatus* was able to sustain mitochondrial capacity up to 30 °C, by compensating the increase of proton leak via a sharp increase of ATP production (Figs. 4, 5). Consequently, increasing rates of mitochondrial ATP production, is a potential adaptive strategy to compensate for a temperature driven loss of cellular energy, as opposed to modulating proton leak itself. This is in line with findings for cold and warm adapted porcelain crabs, where differences in thermal sensitivity of cardiac cellular performance, were not due to changes in membrane fluidity but increased ATPase activity in the warm adapted species^69^. In contrast, fish can modulate proton leak directly in response to environmental temperature, by adjusting membrane lipid composition^70^ or uncoupling protein expression^71,72^. As a result, compensatory increase of ATP production in tropical spiny lobster may extend thermal limits of cellular energy production, but at a higher metabolic cost^73^, which could be a critical shortcoming in face of ongoing ocean warming.

Further, the steep decline of ATP production and mitochondrial function, beyond 30 °C in tropical *P. ornatus*, indicates a small margin to compensate for cellular proton leak (Fig. 4c). This decline occurred far closer to optimal temperatures for growth then for temperate *J. edwardsii* and subtropical *S. verreauxi* (Fig. 5). Consequently, tropical spiny lobster have a much decreased margin between optimal and acute critical cellular cardiac temperatures compared to spiny lobster adapted to colder climates. This is in agreement with other tropical adapted species that show narrower thermal tolerance limits than species from temperate latitudes, making them more vulnerable to temperature stress^74^. In addition, a minor drop of heart rate and oxygen consumption at 31 °C and a significant decline of pseudo cardiac output at 33 °C in *P. ornatus* (Fig. 2), indicate a direct response of cardiac performance to the parallel decline of ATP production beyond 30 °C (Fig. 4c). The recovery of heart rate and oxygen consumption at 35 °C (Fig. 2) suggest short term buffering in cardiac tissue such as by cellular ATP stores (arginine phosphate^75^) or anaerobic ATP production^76^. Sustaining cardiac function and thus blood circulation anaerobically for short periods, could be an intriguing strategy to prevent systemic oxygen collapse under acute heat stress. This interesting aspect remains to be tested in future studies together if similar patterns occur for *J. edwardsii* and *S. verreauxi*, beyond the temperatures tested in this study.

Finally, the larger contribution by mitochondrial complex II to mitochondrial energy turn-over in *P. ornatus* compared to *J. edwardsii* and *S. verreauxi* (Fig. 6), indicates a temperature dependent shift in mitochondrial ATP production and efficiency of ATP synthesis. Mitochondrial complex I and II are essential to convert substrate bound energy into a proton-motive force that drives ATP production. Temperature has shown to affect the balance between both complexes resulting from declining complex I activity such as in crabs^49^ or cuttlefish^77^. This may reflect differential thermal stabilities of different complexes or changes in substrate generating pathways^49^. Most often complex I appears to be the most susceptible to stress^78^. In addition, complex I and II may differ in their kinetic rates. While complex I electron transport chains translocate five protons per electron and complex II moves three, complex II may sustain greater rates of proton translocation^79^ and also provide greater stability for thermally challenged animals, but at the cost of less efficient substrate use. Although differential thermal optima of mitochondrial complexes may limit total ATP productivity – and therefore cardiac capacity to contract - at a particular temperature, it may also enable mitochondria to extend the upper thermal range at which mitochondria can operate, as it is the case for *P. ornatus*. A temperature dependent shift of complex I and II ratios thus suggests a cellular strategy to extend thermal tolerance of cardiac performance towards higher temperatures.

Overall, our data highlight that declines of cellular energetic function may contribute to whole animal performance decline beyond optimal temperatures. Although, likely not the only limiting mechanisms, it provides a mechanistic pathway to better understand thermal tolerance in spiny lobsters and their ecological response to acute or chronic warming. For example, critical temperatures range from 27 to 30 °C (based on aerobic scope) for *S. verreauxi* puerulus^15^, which aligns well with our findings of declining cardiac cellular function at (or below) 30 °C (Figs. 4, 5b) and their naturally observed thermal range of 12–28 °C^80^. If long term warming and acute thermal extremes enter those critical ranges as projected for the northern range of *S. verreauxi*^15^, populations may shift their distribution to cooler waters. This in fact, is strongly supported by an increasing abundance of *S. verreauxi* in temperate South-East Australian waters^81^. While tropical *P. ornatus* (T_crit_ > 31 °C ^82,83^,) may re-distribute to cooler latitudes as well, temperate *J. edwardsii*, (local thermal range of 10.8–17.5 °C, T_crit_ ~18–22 °C^84,85)^ may face compression of its natural distribution range due to the lack of suitable poleward habitat (Fig. 1). In contrast, vertical distribution shifts to deeper cooler waters are unlikely if ecological necessities such as prey and suitable habitat become unavailable.

Some elements of this study limit interpretation and warrant further research. First, since animals needed to be preserved for mitochondrial experiments, we were unable to assess critical (lethal) temperatures. Future studies would need to address the exact sequence of failure of oxygen consumption, heart rate and cardiac output at critical temperatures and assess how this aligns with the patterns observed for mitochondrial function. Second, stroke volume was estimated based on peak height of the reflective infrared signal of the photoplethysmographs. Although this is a valid proxy, it only estimates relative changes rather than absolute stroke volume or selective haemolymph distribution, which could be obtained with a pulsed-Doppler flow meter^55^.

Future research may investigate how cellular efficiencies and critical temperature differ between different types of aerobic tissues combined with whole animal calorimetry to assess overall state of energy leak^86^. Furthermore, it is unclear whether acclimation due to seasonal variation or chronic temperature increase will modulate thermal sensitivity of proton leak or ATP production. It is further desirable to test whether oxygen supply from the gills compromises oxygenation of cardiac tissue in lobsters when temperatures increase and at which critical oxygen levels mitochondrial function declines.

## Conclusion

This study assessed thermal sensitivity in spiny lobsters species to identify functional links between the cellular, organ and whole animal level that contribute to performance decline beyond optimal temperatures. We identified universal patterns between species of spiny lobsters irrespective of their distinct geographical and climatic origin, marked by 1) an increasing divergence between increasing overall oxygen demand but plateauing or declining cardiac performance with increasing temperatures and 2) a parallel decline of cardiac mitochondrial energy production due to increasing proton leak and limited compensation by ATP production. In contrast to temperate and subtropical lobsters, tropical spiny lobsters displayed smaller thermal margins and functional shifts in mitochondrial energy production. We conclude that thermal sensitivity of mitochondrial energy production can directly affect performance of critical organs such as hearts, and thus contribute to defining thermal tolerance in spiny lobsters. An appropriate understanding of the functional cascades driving physiological thermal stress will help to improve forecasts of spiny lobsters’ fitness in response to changing temperatures.

## Material and Methods

### Animals

Study animals comprised three species of Australian spiny lobsters of distinct climatic origins. Cool-temperate southern rock lobster (*Jasus edwardsii*) were caught from the Taroona marine research reserve at Crayfish point, Tasmania, using baited lobster pots from 5–9^th^ February 2018. F1 generation individuals of subtropical eastern rock lobster (*Sagmariasus verreauxi*) and tropical ornate lobster (*Panulirus ornatus*) were obtained from the Institute for Marine and Antarctic Studies (IMAS) onsite aquaculture facility in Taroona, Tasmania. Parental animals of *S. verreauxi* originated from wild caught post larvae at the southern extent of the distribution range at Bicheno, Tasmania in 1999, using a settlement collector, and *P. ornatus* from the Torres Strait regions of Queensland by diving.

The three species of spiny lobsters span the entire latitudinal coastline of Australia. Their distribution ranges from southern Victoria, around Tasmania and across South Australia into Western Australia as well as New Zealand waters for *J. edwardsii*, along the east Australian coastline between Brisbane and the North East coast of Tasmania, including the northern waters of New Zealand for *S. verreauxi*, and the northern Australian coastline from Brisbane to northern West Australia for *P. ornatus* (Fig. 1). All three species live at depths ranging from 5–200 metres at the Australian continental shelf  ^8^. Natural temperature ranges are ~10.8–17.5 °C for *J. edwardsii* (Ion Pot, Tasmania^87,88^), ~12–28 °C for *S. verreauxi*^80^ and ~25–30 °C for *P. ornatus*^89^.

*Jasus edwardsii* were held at 13.3 °C (±SD 0.3 °C) outdoors, in a single coated glass-fibre tank (W × L × H in cm, 205 × 205 × 90), filled with 2.9 m^3^ untreated and aerated flow-through sea water, at a day/night cycle ranging from 11/13 to 14/10 hours. Due to aquaculture holding restrictions and maintenance of water temperatures, *S. verreauxi* and *P. ornatus* were held indoors, in two separate coated glass-fibre tanks (W × L × H in cm, 205 × 205 × 90) at 21.0 °C (±SD 0.1 °C) and 28.0 °C (±SD 0.2 °C) respectively, filled with 2.9 m^3^ filtered and ozone treated, flow-through sea water, at a 14 hour day/10 hour night cycle. Previous testing showed that water quality parameters such as nitrate, ammonia or heavy metals were below critical levels at flow through rates of 1500 L/h and therefore were not tested during holding. Tanks were equipped with off-ground artificial oyster mesh shelters and cleaned every second day to remove food scraps and sediments. Lobsters were acclimated to holding temperatures for at least three months prior to experimentation. All lobster were fed daily with live blue mussels or frozen squid. Weights of lobsters averaged 1030 g (95% CI 909–1153 g, *n* = 6) for *J. edwardsii*, 975 g (95% CI 889–1060 g, *n* = 7) for *S. verreauxi*, and 876 g (95% CI 803–948 g, *n* = 8) for *P. ornatus*.

Animal ethics were not required. Tropical lobsters were handled and prepared within onsite quarantine facilities according to local biosecurity restrictions and legislation (Tasmanian Government Special Authority, SA 18–75).

### Respirometry

**Figure 7:**
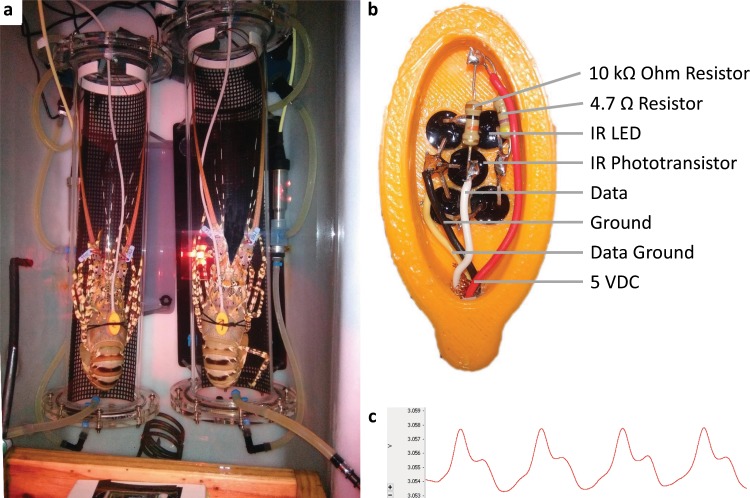
(**a**) Respirometry setup, (**b**) heart rate logger and (**c**) heart rate raw signal of spiny lobster.

Whole animal oxygen consumption was measured using two intermittent flow respirometers^90^. Prior to respirometry, lobsters were collected from holding tanks with a scoop net and a wet towel covering their eyes to reduce transfer stress. Following the fixation of heart rate loggers, individuals were transferred into cylindrical 10 L respirometers (L × D in cm, 66 × 15), with tail first to prevent blocking and injury due to the lobster’s guarding posture. Lobsters could gain traction to oyster mesh added to the bottom of the respirometer, held in place with an open cut piece of plastic pipe (Fig. 7a)^91^. Chambers were sealed within two minutes after addition of lobsters. Oxygen concentration was measured continuously using a fibre optic two-channel oxygen meter (HQ40d, Hach, USA), with oxygen probes positioned into the external recirculation loop. Re-circulation pumps (Quite One® Pro 1200, Lifegard Aquatics, USA) provided continuous mixing of water within respirometers at a flow of 1200 L/min. Following a six-minute respiration cycle, flush pumps (Compact + 2000, Eheim, Germany) re- oxygenated chambers at a flow of ca. 1500 L/min for eight minutes, using a time controlled digital recycling timer (DRT-1, Sentinel, USA). The two adjacent respirometers were housed in a buffer tank (W × L × H in cm, 102 × 52 × 50), filled with 190 L filtered and ozonated flow-through sea water at a flow rate of 130–150 L/h. An air stone ensured homogenous mixing in the buffer tank and supply of aeration. The experimental setup was covered with black building foil to prevent visual disturbance and illuminated with an LED flood light set to yellow at low intensity. Lights were permanently turned on during experiments to reduce spontaneous activity of the nocturnal lobsters^92^. After each experiment, the respiration setup was cleaned and flushed with fresh water.

Water temperature was increased from ambient starting temperature using a 2000 Watt titanium heater (Istra Elements, Australia) modified with a programmable PID controller (SmartPID, Arzaman, Italy). Following an acclimation time within the respirometers of minimum 11 hours, temperatures were ramped from 14–23 °C for *J. edwardsii*, 20–28 °C for *S. verreauxi*, and 28–35 °C for *P. ornatus* at a heating rate of ca. 2.3 °C per hour. Respiration was measured for one hour at each temperature ramp. After completion of the final heating ramp, temperatures returned to initial acclimation temperature at about 3 °C per hour and lobsters were returned to the holding tank for at least 24 hours before further use for mitochondrial experiments, with the exception of four specimens of *P. ornatus*, which were used after five hours acclimation. Note that experimental temperature ranges were not chosen to cover the full natural thermal range of the respective species, but to test effects of elevated and close to critical temperatures on lobster metabolism and cardiac performance.

Respiration was calculated as in Svendsen *et al*.^93^. Oxygen saturation in respiration chambers decreased to a minimum of 87% (95% CI 85–89%) on average, and never fell below 75%.

Individual body volume of lobsters was accounted for in *M*O_2_ calculations and measured as the volume overflow in ml after adding lobsters to a water levelled container. Body density did not differ significantly between species (ANOVA, *F*_(2, 18)_ = 2.01, *P* = 0.163) and averaged 1.33 g/ml (95% CI 1.25–1.41 g/ml). Background respiration was recorded before and after each experiment and accounted for 4.5% (95% CI 3.3–5.7 g/ml) on average relative to standard metabolic rate (no significant differences between species, ANOVA, *F*_(2, 18)_ = 0.36, *P* = 0.701). Preliminary tests confirmed appropriate mixing of water in the chamber, the lack of leaks (i.e. dye test) and appropriate flush/respiration cycles.

Moulting can significantly increase standard and routine metabolic rates^94^. We accounted for this by measuring the Brix index^95,96^ of 100 µl haemolymph from each lobster, following centrifugation at 10.000 g for 3 min, using a refractometer (Hanna HI96801, Hanna Instruments, Australia). The Brix index did not show significant interaction with metabolic rates (ANOVA, *F*_(1,14)_ = 1.178, *P* = 0.296), indicating that our metabolic data were not affected by the moulting stage of the experimental lobsters.

### Heart rate

Heart rate of lobster was recorded simultaneously during oxygen consumption rate measurement using a self-assembled infrared photoplethysmographs (Fig. 7b) analogue to previous sensors used for decapod crustaceans^36,97^. This comprised four infrared LEDs (SFH 4544, 950 nm, T-1 3/4, 5 mm, Osram, Germany) arranged around one infrared phototransistor (SFH 313 FA-2/3 20°, 740–1080 nm, T-1 3/4, 5 mm, Osram, Germany) glued into a 3D printed ABS plastic casing (3D model in Supplementary File S). The electronics were water proofed with hot glue, the lid fixed with superglue and the plastic surface welded with acetone. The photoplethysmograph sensors was powered from a 5 VDC laptop USB port and connected to a 2-channel A/D recorder (PowerLab15T Model ML4818, AD Instruments, New Zealand) via a four-wire cable to record the analogue voltage signal from the phototransistor (Electrical circuit diagram in Supplementary Fig. S). A plastic cable gland in the lid of the respiration chamber secured and sealed the cable.

Prior to the addition to the respiration chambers, heart rate loggers were positioned dorsally above the heart and fixed with two cable strips. Dental wax between the logger and the lobster’s carapace reduced slipping and signal disturbance by water movements. Short test recordings during positioning and just prior to the beginning of the respiration experiment confirmed a viable heart rate signal. A wet towel covering the eyes and antennae of lobsters reduced handling stress during the attachment procedure.

The analogue raw signal from the photoplethysmographs (Fig. 7c) was analysed using LabChart 8 Pro (AD Instruments, New Zealand), applying the following settings to calculate. beats per minute: 500 mV voltage range, 400/s recording frequency, triangular smoothing with 151 samples, 5 Hz low pass filter and cyclic measurement rate with general sine shape and a minimum peak height of 0.18 SD. Peak height (in volts) was extracted as an indicator of cardiac stroke volume, assuming that decreasing fluid content (cardiac volume), increases signal intensity, due to reduced absorbance by water within the ventricle. A proxy for cardiac output was calculated as the peak height multiplied by heart beat rate (per minute) and denoted as pseudo cardiac output.

### Mitochondrial function

For the analysis of cardiac mitochondrial function, the same animals used for respiration and heart rate experiments were used. Lobsters were removed from holding tank and anaesthetized by chilling them in 0 °C sea water. Once lobsters stopped responding to physical contact (~10–20 min), the carapace was opened dorsally by two longitudinal parallel cuts above the gill chamber and carefully cut off from connective tissue and muscle.

The heart was immediately dissected and immersed in 2 ml modified ice-cold relaxing buffer (in mmol/L: 2.77 CaK_2_EGTA, 7.23 K_2_EGTA, 5.77 Na_2_ATP, 6.56 MgCl_2_·6H_2_O, 20 taurine, 20 imidazole, 0.5 dithiothreitol, 50 MES, and 400 KCl, pH 7.1 at 0 °C, final osmotic concentration was 960 mOsm). The heart tissues were cut into small pieces and stored in relaxing buffer on ice until required and excess tissue snap frozen in liquid nitrogen and stored at −80 °C. Approximately 100 mg of tissue was accurately weighed into a micro-centrifuge tube with a 10 fold w/v volume of respiration media (in mmol/L: 0.5 EGTA, 3 MgCl_2_, 100 K-lactobionate, 20 taurine, 10 KH_2_PO_4_, 20 HEPES, 200 sucrose, 250 KCl and 1 g/L BSA, pH 7.24 at 20 °C; Gnaiger *et* al. (2000)), and then homogenized using a tissue homogenizer (Dremel, 985370–395, USA). Homogenate (totalling 10 mg of tissue for *J. edwardsii* and *S. verreauxi* and 5 mg tissue for *P. ornatus*) was added to each chamber of calibrated Oroboros O2K oxygraphs (Oroboros Instruments, Innsbruck, Austria). Tissue homogenates were used as this permitted 1) rapid analysis of tissues, 2) a measure of the respiratory capacity of the wet mass of heart tissue, 3) decreased optical disturbances that permeabilised fibres can produce, and 4) rapid uptake of the mitochondria membrane potential indicator (TMRM). Fresh homogenate was used for each experiment.

Respiration and membrane potential of cardiac tissue homogenate was measured simultaneously in two replicate 2 ml glass chambers, using two Oroboros O2K oxygraphs, each set at two different temperatures, which were specific for each species. Samples of* J. edwardsii* were assayed at 15, 20, 25 and 30 °C, *S. verreauxi*, at 20, 25, 30 and 35 °C and *P. ornatus* at 25, 30, 35 and 37.5 °C. Temperatures were alternated among oxygraphs to account for possible differences in sample holding times and oxygraphs. Mitochondrial membrane potential was measured using fluorimeters, attached to the transparent front of each glass chamber, each using green excitation LEDs (530 nm) with red (580 nm long-pass filters). The fluorescent dye Tetramethylrhodamine (TMRM, Thermofisher Scientific) was titrated into each chamber in two injections of 125 µmol/L (totalling 250 µmol/L TMRM). These were used to calibrate the TMRM signal, which was allowed to settle prior to addition of mitochondrial substrates. The mitochondrial substrates malate (2 mmol/L) and glutamate (10 mmol/L) were then added to elevate membrane potential and provide a measure of leak state respiration, which results from proton conductance (leakage) across the inner mitochondrial membrane. ADP (2.5 mmol/L) was then added to initiate oxidative phosphorylation supported by mitochondrial complex I. Oxidative phosphorylation commencement also partially depolarizes the mitochondrial membrane potential. Oxidative phosphorylation was further enhanced by addition of the amino acid substrate proline (10 mmol/L), and then succinate (10 mmol/L). Proline is oxidised by proline dehydrogenase and *in vivo* the ensuing products should also support mitochondrial complex I and complex II. Succinate is oxidized by mitochondrial complex II. The addition of proline and succinate therefore elevate oxygen flux *JO*_2_. Oligomycin was then added (2.5 µmol/L) to induce a maximal leak respiration state LEAK_max_, which coincides with a high mitochondrial membrane potential that is higher than that with single substrates. Titrations (1–3 additions, dependent on temperature) of the un-coupler carbonyl cyanide m-chlorophenyl hydrazone (CCCP 0.5 µmol/L) was then added to measure the maximum capacity of the electron transport system (ETS) and to depolarize mitochondrial membranes. Unless stated, all chemicals were obtained from Sigma-Aldrich (St. Louis, MO, USA).

Simultaneous to mitochondrial membrane potential, mitochondrial respiration was measured in the same chambers, using high resolution polarographic oxygen sensors, as mass-specific oxygen flux [pmol O_2_/(sec mg wet weight)]. Oxygen flux *JO*_2_ was calculated in real time as the negative time derivative of the oxygen concentration using Oroboros DatLab Software V 7.1. (Oroboros Instruments, Innsbruck, Austria).

In addition, we estimated the total ATP formed in maximal oxidative phosphorylation through several assumptions. We assumed that the measured mitochondrial oxygen flux *JO*_2_, transfers to ten protons for complex I/NADH derived electrons and six protons for complex II derived electrons being pumped into the intermembrane space. We further assume that proline, which is FAD^+^ linked at proline dehydrogease (ProlDH, only), or FAD^+^ and NAD^+^ linked (ProlDH and complex I and II) is fully oxidized. This results in respective substrate ATP:Oxygen (P:O) ratios of approximately 2.5 and 1.5 for complex I and complex II respectively, and 1.5 and 2.15 for proline with incomplete or complete oxidation of the products of proline oxidation respectively. We assumed that there are eight subunits in the ATP synthase C-ring and one rotation forms three ATP, and that increases in oxygen flux *JO*_2_ resulting from substrate addition, were proportionate to the electron flow through each respiratory complex, and that there was no reallocation or restriction of the Q-pool. We note that if the proportion of electrons flowing from different complexes changes with substrate addition (i.e. due to the addition of FAD^+^ linked substrates after complex I linked substrates), we will overestimates ATP synthesis rates.

Mitochondrial membrane potentials were not calculated, or reported as relative fluorescence. This was because the mitochondrial volume within homogenates was unknown and relative fluorescence would not allow comparisons among species. Here we report TMRM data as the amount of probe imported or expelled from mitochondrial with respective polarization and depolarization, with units of nmol TMRM per mg of tissue. This was calculated from measuring the differences in signal before or after addition of specific substrates, oligomycin, and on uncoupling with CCCP. This permitted calculation of the amount of TMRM taken up in oxidative phosphorylation and at maximum leak respiration.

To incorporate the oxygen flux *JO*_2_ and TMRM data we considered that the amount of imported TMRM is dependent on oxygen flux *JO*_2_ in specific states, and this represents a form of work to maintain a membrane potential. Therefore, we converted the oxygen flux *JO*_2_ into Watts assuming that the reduction of oxygen to H_2_O equals 20 kJ/mol, and in this way we explored the amount of TMRM taken up per unit mitochondrial work (energy expended) in performed maximum oxidative phosphorylation. This represents the demands where maximal demands are placed on the oxidative phosphorylation system, and also in maximal leak respiration state LEAK_max_, as this provides insights to the membrane integrity while under high load.

### Data analysis

All data statistical data analysis was performed using R statistical software^98^ and RStudio^99^.

Temperature ramp data (Figs. 2–5) were analysed using repeated-measures ANOVA followed by Tukey Posthoc test with Bonferroni correction, to compare data between the starting temperature and data at successive temperature ramps. If data failed ANOVA assumptions for normality and homogeneity of variances, a non-parametric Kruskal-Wallis test was applied followed by Dunn test for multiple comparisons and “Benjamini-Hochberg” correction to decreases type I error (i.e. false positives). The relation between pseudo cardiac output and oxygen consumption (Fig. 3) was analysed, by comparing two linear regression models with and without species as co-factor. Complex II substrate use was compared between species on data pooled across temperatures (Fig. 6), using non-parametric Kruskal-Wallis test, followed by Dunn test for multiple comparisons with “Benjamini-Hochberg” correction.

Standard metabolic rate was calculated as the mean of the lowest 10% of values for the initial acclimation period, with outliers excluded below ±2 SD of the mean^27^. Routine metabolic rates for each temperature ramp were calculated as the average respiration of a ramping level each lasting for one hour.

Heart rate data were extracted specific to each respiration cycle used for metabolic rate calculations, excluding data outside this range (e.g. ramping periods, flush cycles). To reduce the impact of signal noise, due to animal movements, heart rate data were filtered choosing binned regions with low signal variance within each six minute respiration interval.

## Supplementary information

Supplementary information.

Supplementary information.

## Data Availability

The datasets generated during the current study are available in the Figshare repository, doi 10.6084/m9.figshare.9162218.
