# Supplementary material for: Thermal sensitivity links to cellular cardiac decline in three spiny lobsters
Source: Sci Rep. 2020 Jan 14;10:202. doi: 10.1038/s41598-019-56794-0 (PMC6959275; doi:10.1038/s41598-019-56794-0)
Supplement: Supplementary file 2 — Supplementary information. [file 41598_2019_56794_MOESM2_ESM.pdf]

Supplementary Information

Thermal sensitivity links to cellular cardiac decline in three spiny lobsters

Michael Oellermann[1,*], Anthony Jr. Hickey[2], Quinn P. Fitzgibbon[1], Greg Smith[1]

[1] Fisheries and Aquaculture Centre, Institute for Marine and Antarctic Studies (IMAS), University of Tasmania, Hobart, Tasmania, 7001, Australia

[2] School of Biological Sciences, University of Auckland, Auckland, New Zealand

* michael.oellermann@utas.edu.au

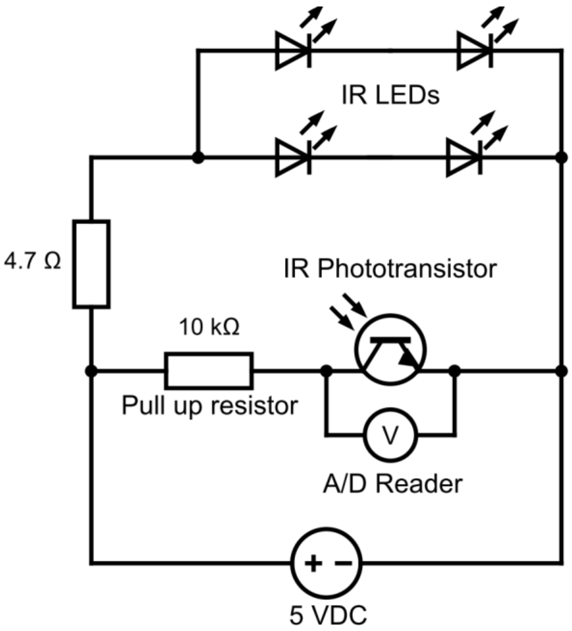

Supplementary Figure S2: Electrical circuit diagram for self-assembled photoplethysmographs to measure heart rate in spiny lobsters
